# Closing the gender gap in entrepreneurship: The importance of skill variety

**DOI:** 10.1371/journal.pone.0270976

**Published:** 2022-07-08

**Authors:** Alexander Krieger, Jörn Block, Michael Stuetzer, Martin Obschonka, Katariina Salmela-Aro

**Affiliations:** 1 Department of Industry, Baden-Wuerttemberg Cooperative State University, Mannheim, Germany; 2 Department of Economic and Social Sciences, Trier University, Trier, Germany; 3 Witten Institute for Family Business, Trier, Germany; 4 Erasmus School of Economics, Erasmus University Rotterdam, Rotterdam, Netherlands; 5 Faculty of Economic Sciences and Media, Ilmenau University of Technology, Ilmenau, Germany; 6 Amsterdam Business School, University of Amsterdam, Amsterdam, Netherlands; 7 Department of Educational Sciences, University of Helsinki, Helsinki, Finland; Wroclaw University of Economics and Business: Uniwersytet Ekonomiczny we Wroclawiu, POLAND

## Abstract

Given that skill variety is widely regarded a key component of entrepreneurial human capital, gender differences in entrepreneurship could be rooted in the formation of such skill variety. Analyzing 12-year longitudinal data following 1,321 Finnish adolescents into adulthood, we study whether gender differences in skill variety open up early in the vocational development of entrepreneurs vs. non-entrepreneurs, thereby contributing to the persisting gender gap in entrepreneurship in adulthood. Specifically, structural equation modeling was used to test and compare the mediating effect of early skill variety in adolescence vs. education- and work-related skill variety in early adulthood on the gender gap in entrepreneurial intentions in adulthood. We find that education- and work-related skill variety indeed operate as an obstacle for women entrepreneurship, despite women outperforming men in early skill variety in adolescence. Hence, we identify a critical turning point in early adulthood where women fall behind in their development of entrepreneurial human capital.

## Introduction

Research found a (historically) persisting gender gap in entrepreneurial intentions and activities, with males showing a somewhat higher likelihood of forming entrepreneurial intentions, and entrepreneurial activity respectively [1,2]. Several explanations have been brought forward to explain this gap. These explanations relate to gender differences in social [3] and human capital [4,5] as well as gender differences in access to financial capital [6,7] and personality [8].

Our paper focuses on gender differences in (entrepreneurial) human capital and proposes differences in skill variety as an additional, hitherto not examined explanation for the gender gap in entrepreneurial intentions. Lazear [9], who introduced the concept of skill variety into the entrepreneurship literature, argues that entrepreneurs should be good at a variety of tasks to make the business succeed. Following this logic, entrepreneurship is essentially a multi-task phenomenon and requires a balanced set of skills to set up and manage a new venture [10]. This skill variety approach has received broad empirical support–both in the prediction of entrepreneurial behavior and success [11–14]. So if skill variety is such a critical component of entrepreneurial human capital, what is its role in the gender gap in entrepreneurship?

Interestingly, there is first preliminary empirical evidence suggesting that, on average, woman may develop lower skill variety than men do [15,16]. Our paper builds on this line of research and investigates whether and to what extent gender differences in entrepreneurial skill variety indeed exist and open up early in vocational development, thereby laying the foundations for the gender gap in entrepreneurial intentions in adulthood. We focus on two interrelated research questions: (1) To what degree does skill variety provide an explanation as a mediator for the gender gap in entrepreneurial intentions? 2) How does the mediating effect of skill variety in the relationship between gender and entrepreneurial intention change over the educational trajectory?

Our paper links the entrepreneurial intention literature concerned with the explanation of systematic gender differences [2] with the human capital concept of skill variety, which is an emerging topic with promising research findings so far [17]. It has been argued, that the concept of skill variety arguably covers the nature of the entrepreneurial task better than more traditional human capital variables such as length of education and work experience [13]. Our paper extends the human capital explanation for the gender gap in entrepreneurship [4,5] by including age-appropriate markers of skill variety in a person’s vocational development across adolescence and young adulthood as potential mechanisms. Next to this, the empirical results of our paper may also have practical implications for entrepreneurship educators and policy makers. Educating skill variety might improve by making changes in school and university curricula and by introducing special entrepreneurship education programs designed to improve the various facets of skill variety.

We investigate our research questions with a 10-year longitudinal dataset following 1,321 Finnish adolescents into adulthood. Our method of choice is structural equation modeling which we use to test and compare the mediating effect of early skill variety in adolescence vs. work-related skill variety in adulthood on the gender gap in entrepreneurial intentions. In line with previous research [18–20], our results show a clear gender gap in entrepreneurial intentions. According to our results, women have considerably less interest and plans to pursue an entrepreneurship career. Our data also show that women exhibit less *educational* and *work skill variety*, while they report higher scores for *school interest variety*, which we measure at an earlier stage in their educational career. This means that women have more skill variety than men at an early age, but this trend reverses and men have more skill variety at a later age. All three skill variety measures predict entrepreneurial intentions. Finally, we find evidence that both e*ducational* and *work skill variety* mediate part of the gender gap in entrepreneurial intentions.

Our study makes two contributions to the entrepreneurship literature. First, it contributes to research concerned with gender aspects in entrepreneurship, particularly to the human capital explanation for the gender gap in entrepreneurship [4,5]. Second, our study contributes to the skill variety or jack-of-all-trades view of entrepreneurship [9,14,21–25] and its applications for practical research questions such as gender disparities in entrepreneurial tendencies. Our paper builds on previous findings reported in [14]. They already showed with the same data set that skill variety in general predicts entrepreneurial intentions. In the present paper we test gender differences in skill variety as potential mechanism in the gender gap in entrepreneurial intentions. The data and syntax to analyze it are available under https://osf.io/4fh8u/ under a Creative Commons License (CC BY).

The remainder of the study is organized as follows. Section 2 introduces our conceptual model and develops hypotheses about skill variety as a mediator explaining the link between gender and entrepreneurial intentions. Section 3 presents the data, methods and variables. Section 4 explains the empirical strategy and shows the results of the hypothesis testing. We discuss the results and their implications for entrepreneurship theory and practice in Section 5.

## Conceptual model and hypotheses

### Gender gap in entrepreneurship and existing research on gender and skill variety

There is a broad consensus about the existence of a gender gap in entrepreneurial career choice [1,26,27]. Research showed that men differ from women in that they often report, on average, stronger entrepreneurial intentions and also a higher likelihood of early-stage entrepreneurship activity [1,19,28].

What do we know about the drivers behind the gender gap in entrepreneurship? The gap is widely acknowledged as a multi-causal phenomenon [1,8,19]. The most discussed mediating factors explaining the gender gap are social capital [3,29], financial capital [6,7,30] and human capital [5,31]. We will theorize below in greater detail how these factors contribute to the gender gap in entrepreneurial intentions, but put a greater emphasis on human capital because our paper focus is on skill variety as a potential mediator explaining the gender gap in entrepreneurial intentions. In the present study, we argue that, on average, men and woman differ in skill variety and this difference mediates the relationship between gender and entrepreneurial intention. The respective jack-of-all-trades theory [9] posits that being an entrepreneur requires skills and knowledge in several fields and that people having a varied set of skills and knowledge should therefore be more successful entrepreneurs. There is some empirical evidence that female entrepreneurs have less skill variety compared to male entrepreneurs [15,31,32]. Further, using an all-female sample, [33] found evidence that skill variety is important for the entry decision of women. [32] reported skill variety to partially mediate the gender gap in firm innovation.

### Theorized mechanisms on linking gender and entrepreneurial intentions in adulthood

In this section, we theorize on how social capital, financial capital and human capital explain the gender gap in entrepreneurship. Because our dependent variable in the empirical section is entrepreneurial intentions and not entrepreneurial behavior, we emphasize the intention aspect in our theorizing. Entrepreneurial intentions precede behavior [34,35], because people invest a considerable amount of time and financial resources in the activity. Entrepreneurship is something people plan or choose to do [36]. In other words, there is a certain readiness to engage in entrepreneurship [37,38] which manifests in entrepreneurial intentions as a predictor of entrepreneurial behavior [34,35,39].

*Social capital* was originally developed in sociology and deals with individuals’ social relations as well as possible benefits or drawbacks resulting from these relations [40–42]. Regarding entrepreneurship, social capital is important because it 1) helps nascent entrepreneurs to overcome substantial resource constraints [43] and 2) provides access to novel information and granted feedback about business strategies, which is particularly important to shape entrepreneurial intentions and behavior [44–46].

Literature on female entrepreneurship and social capital shows clear differences in the individual social networks between the sexes. Women view social networks differently than men [29,47]. In social networks, men see what they may gain from it, while women place a focus on responsibilities and obligations [48]. Moreover, women rather involve family members within their business activities, while men also rely on external partners, often other entrepreneurs, who are important role models [3,49]. The structure of male networks with more business contacts enables men to access more resources and gain better feedback regarding the idea of setting up a firm compared to woman, which should ultimately result in stronger entrepreneurial intentions for men.

*Financial capital* is crucial during the start-up process because the business does not yet generate funds [7,30]. Women entrepreneurs often start with significantly lower levels of financial capital than men [30,50]. Research has found different areas of the financing process to pose obstacles women have to overcome in the process of setting up a new venture [51], even though women tend to found smaller, less growth-oriented firms in industries where there is less startup capital needed, for example in the retail and service sector [52–54]. There is evidence that women might be disadvantaged in raising start-up capital [6,55], as well as in providing guarantees, such as personal assets or track records [56,57]. Research has also found that women apply less for debt capital than men [58,59]. Fielden et al. [60] confirm this result and further report that women have lower beliefs in getting credit. With regard to the obstacles women face in financing future ventures and their lower belief in getting external finance, we argue that this can discourage female entrepreneurial intentions.

*Human capital* is defined as knowledge and skills that are acquired through schooling, on-the-job training and other kinds of experience [61]. Human capital theory predicts that investment in knowledge and skills leads to individual increases in cognitive abilities, which in turn leads to more productivity and efficiency in potential activities [61,62]. Human capital is important for entrepreneurship for at least two reasons. First, it helps spotting and developing entrepreneurial opportunities [63]. Second, skills and knowledge are essential for managing the complex task of setting up and running a business [64]. Despite theoretical acclaim, the meta-analysis from Unger et al. [65] has cast some doubt on the importance of human capital for entrepreneurship by reporting surprisingly low correlations especially between general human capital measures (e.g., formal education or years of working experience) and entrepreneurship. Such general human capital measures are widespread in entrepreneurship research [66]. More specific or task-related human capital measures such as prior start-up experience or managerial experience fare better in empirical analyses [65].

Literature on female entrepreneurship and general human capital shows mixed evidence. There has been a gender difference in average years of education until the 1970s [67,68]. However, nowadays, in the developed economies, education levels have converged between male and female, thus this is not a compelling explanation anymore for the gender gap in entrepreneurship [31,69]. Potentially more important are gender differences in work experience. Despite an increase in the labor force participation over the last decades, women are still more likely to have worked part-time and fewer years overall [70,71]. Especially in business and management as well as technical functions, men have more practical experience [5,72]. It should be noted that individuals working in managerial, technical and craft functions are more likely to become entrepreneurs [73].

The higher levels of work experience for men can contribute to the formation of their entrepreneurial intentions. We base this reasoning on theoretical models in psychology arguing that intentions to perform a task are partly driven by the ability and partly by the belief in the ability to perform this task [74,75]. As abilities rise, so do the belief in the ability, which results in higher levels of entrepreneurial intention for men. In a related paper, Tonoyan et al. [4] provide empirical evidence across 22 countries, that especially the combination of woman working in non-managerial positions, female dominated occupations and sectors explain roughly 30% in variation in the perceived ease to start a firm.

Taking the arguments from the literature on social capital, financial capital and human capital together, we hypothesize:

*H1*: *There are gender differences in entrepreneurial intentions in adulthood with men shower a higher likelihood for such intentions than women*.

### Gender and skill variety in the vocational development between adolescence and adulthood

Our central hypothesis in our paper focuses on potentially influential gender differences in skill variety. As noted above, task-related human capital, in contrast to broad, general human capital [9,13,65], is particularly relevant for entrepreneurial careers because it directly refers to the various entrepreneurial tasks. To account for the entrepreneurial task profile, Lazear [9] introduced the concept of skill variety for entrepreneurs. In the spirit of Lazear, we define skill variety as having a varied set of skills and knowledge that are task-specific, and thus highly relevant, for entrepreneurship. Having such a varied set of skills and knowledge should be beneficial for entrepreneurs because entrepreneurs deal with these many different tasks within their enterprises such as developing business models, talking to customers, negotiating with suppliers or hiring and instructing employees. A number of papers show the significance of skill variety for entrepreneurship choice and success [9,22–25,76].

In the following, we will argue that there are pronounced gender differences in skill variety and that this difference can explain part of the gender gap in entrepreneurship. We base our theorizing on gender-specific educational and employment experiences. In general, skill variety can be acquired by studying in different fields, working in different jobs or having a job with a broad spectrum of tasks such as management [9,22,25]. Thus, gender differences in skill variety might be rooted in gender differences, not in educational attainment and labor force participation per se, but in gender differences fields of study and employment history.

Regarding education, there are gender differences concerning the choice of major subjects [77]. Albeit gender differences in the fields of studies have shrunk in recent decades, women are still greatly underrepresented in traditionally male-dominated fields such as mathematics, engineering or physics and greatly overrepresented in traditionally female-dominated fields such as education, health care and humanities [72,78,79]. According to Blau and Khan [80], in the majority of developed countries, around 30% of all graduates in engineering and natural sciences are female and around 70% are male. In contrast, in education, health care and cultural studies, only around 25% of the graduates are male, while around 75% are female.

We argue that women, on average, are less likely to acquire skill variety in their predominant fields of studies. This is because curricula in traditionally female-dominated fields have in general not included technical or such as mathematics or business courses into their curricula [81]. Thus, they often do not acquire greater skill variety. This is even partly rational because graduates from these fields very often sftay in their industries. Take for example, a teacher or a nurse. They are on a predefined career path and seldom switch industries. There is also little self-employment in these industries [82]. Without a subsequent suitable use of skill variety, educational curricula are perfectly matched with job requirements to focus on rather narrow set of skills (e.g. teaching skills and knowledge about a specific subject for a teacher). In contrast, traditionally male dominated fields such as engineering of studies often include non-technical courses. One apparent example are the Master of Business and Engineering. Empirical evidence for this reasoning is provided by [83], who show (with German data) that engineers, chemists and physicists, followed by managers and business consultants, clearly have the highest levels of skill variety.

Regarding work experience, recall that women have in sum worked fewer years and are more likely to be part-time workers [5,70,71]. With limited work experience, it is very difficult to gather variety in their skill set from work. This is because one can arguably do more different things when working full-time and longer years instead of part-time and fewer years. Beyond time restrictions, female-dominated occupations in paid employment (e.g. in education and health care) are more often found in subordinate positions, compared to male-dominated occupations [84,85]. In a subordinate position, one is less likely to acquire entrepreneurial human capital, especially in the management and decision-making process. This means women lack skill variety due to the character of the jobs they hold [69].

Women are also arguably less likely to acquire skill variety by switching jobs. One potential reason for this are gender differences in fear of failure and risk-aversion where women score higher [86,87]. Fear of failure and risk aversion prevent people from trying out new things, e.g. new and different jobs and induce staying with the same job or within the same industry [88–90]. Another reason for less job switching is that women are less locally and timely flexible, due to child and family commitments [91–93]. This limits their choices for potential employers as well as different industries and thus their chances to acquire skill variety through job mobility. This is not to say that women do not switch employers, but their job pattern is more characterized by switching employers in the same industry [94–96], which does not lead to much more skill variety. In addition, women tend to concentrate in more specialized sectors, such as health care or the public sector, that make the accumulation of skill variety difficult [97–100]. This is because especially these sectors includes large, bureaucratic and hierarchical organizations with a pronounced division of labor. Skill variety is rather acquired in small firms [31,101], where there are less predefined job descriptions and therefore less division of labor [102,103].

Taking together the arguments from above on educational and subsequent work segregation between male and female, as well as different preferences in schooling and work, we hypothesize:

*H2*: *There are gender differences in skill variety with men showing a higher likelihood for skill variety than women*.

### Skill variety as human capital driver of entrepreneurial intentions

One of the greatest challenges for an entrepreneur is the heterogeneous nature of the entrepreneurial work tasks. In contrast to most jobs in paid employment, where specialization is an asset [9], self-employed persons often profit most from being just the opposite [15]. By possessing a well-diversified set of skills, a jack-of-all-trades is better equipped to master the variety of challenges of starting-up and running a business [25].

Lazear [9] concluded that a variety in experiences and competencies is a crucial characteristic of self-employed persons. Quite a number of studies have shown that such skill variety increases the probability of becoming an entrepreneur [9,22–24,76]. A diverse educational curriculum as well as working in different functions and for a number of employers have been named likely sources of skill variety [23]. Regarding performance effects most studies report positive correlations of skill variety and different measures of performance. Stuetzer et al. [13] find that skill variety helps nascent entrepreneurs to make progress in the venture creation process and Oberschachtsiek [104] reports that entrepreneurs with a more varied skill set remain in self-employment longer. Bublitz & Noseleit [105] find that skill variety has positive effects on incomes for entrepreneurs but not for employees. However, Åstebro & Thompson [22] find that a varied skill set is related with less income from self-employment.

Given this generally positive relationship between skill variety and entrepreneurship, we argue that people who exhibit variety in skills realize that correlation and are thus more likely to unfold intentions to become an entrepreneur. This reasoning is based again on theoretical models in psychology arguing that intentions to perform a task are partly driven by the ability and the belief in the ability to perform this task [74,75]. Backes-Gellner & Moog [106] provide suitable empirical evidence on this argument by pointing out the correlation between a broad human capital portfolio and entrepreneurial intentions.

Recall that we mentioned the related work of Krieger et al. [14] which based on the same data set already showed that skill variety predicts entrepreneurial intentions. Thus, this hypothesis is not new, but we feel a need to include the hypothesis in this paper to complete the path model from gender via skill variety towards entrepreneurial intentions, and to establish a mediation test.

*H3*: *Skill variety shows a positive relationship with entrepreneurial intentions*.

### Skill variety as a mechanism behind the gender gap in entrepreneurial intentions

Given the argument that women, on average, accumulate less skill variety over time, and that this skill variety, in turn, is a driver of entrepreneurial intentions, we expect skill variety to mediate the relationship between gender and entrepreneurial intentions. We thus formulate the following basic mediation hypothesis:

*H4*: *The gender difference in entrepreneurial intentions is mediated by skill variety*.

Next to this basic mediation hypothesis, we also take a longitudinal perspective and argue that the mediating influence of skill variety in the relationship between gender and entrepreneurial intentions becomes stronger across the educational trajectory. Our argument consists of two steps.

First, we argue that the negative relationship between gender and skill variety grows stronger over the educational trajectory from school via college towards working life. The origins and drivers of gender differences in schooling and work preferences are controversially discussed. One explanation is job market discrimination in traditionally male-dominated jobs [77], such as science, engineering or production [107]. So if there is discrimination or even just expected discrimination in the labor market, this probably has trickle down effects for prior educational choices. A rational response of parents to discrimination in the labor market could be a different treatment or breeding of boys versus girls to shape their preferences to be compatible with the labor market [108]. From a competitive advantage point of view, it makes sense for someone to specialize in those fields where there is least discrimination against oneself and thereby accumulate corresponding human capital [77,109,110]. Thus it is possible that already in school there might be less skill variety for girls and more skill variety for boys because parents anticipate potential labor market discrimination. Later on educational choices in college might follow the same pattern but more accentuated. During work, the gender segregated experiences will be arguably even stronger as women than might face actual discrimination or follow traditional patterns of men being the male breadwinner and women working part time or drop completely out of the labor force due to child and family commitments [91–93].

Second, we also expect the effect of skill variety on intentions to grow stronger across the educational trajectory. This is because variety in school might be of limited relevance for later entrepreneurship while skill variety from different work experiences such as working in different fields like marketing or human resources is of direct relevance for the tasks an entrepreneur faces when setting up and running a business.

We formulate the following longitudinal hypothesis about the strength of the mediating effect of skill variety:

*H5*: *The mediating influence of skill variety in the relationship between gender and entrepreneurial intentions becomes stronger across the educational trajectory*.

## Data and methods

### Data collection

We test the hypotheses in a longitudinal sample of students, covering both an early developmental phase (adolescence) as well as working life. More precisely, we use the (ongoing) Finnish FinEdu study, a data set collected by the University of Helsinki and the University of Jyväskylä, aimed at young adults’ personal goals and concerns in the domains of career, comprising education, work and financial issues [111] and entrepreneurship items (e.g. entrepreneurial intentions, skill variety).

The first wave was raised in 2004, followed by 7 further waves over a span of twelfe years. At the first wave, the respondents’ average age was 16 years, therefore the survey was conducted at school. The students were asked about their permission and University of Jyväskylä gave the ethical permission to Jari-Erik Nurmi when the data was collected for the first time. More generally, the Finnish FinEdu study follows the Helsinki declaration (https://en.m.wikipedia.org/wiki/Declaration_of_Helsinki). Students from two different school tracks (A: lower secondary school, N = 707; B: academic track of upper secondary school, N = 614) were followed through their school lives and early careers [112]. In the present analysis, we combine the data from both school tracks, controlling for school track effects. This procedure has been successfully employed in previous research [113,114]. The respondents’ average age was 28 at the 2016 wave. Here, the study was sent out by mail and completed by phone [113,114]. Looking at the gender distribution, 46% of the participants were male (N = 603) and 54% (N = 708) were female. Here, women are slightly overrepresented, which reflects the overall distribution of students at this educational level [113,115]. In sum, we rely on data collected among 1,321 participants.

For our purpose, we mainly use the data from first wave in 2004 (respondent average age 16) and the wave 6 in 2014 (respondent average age 26) which contain the data regarding skill variety and entrepreneurial intentions. In a robustness check we also use the data from wave 7 from 2016 which contains information on entrepreneurial behavior. Please not that we will not use entrepreneurial behavior as outcome in the model for two reasons. Firstly, the age 28 is too early for many to start a business. At this age a sizeable portion of the respondents is still at university, and those who graduated have just started their first job. Entrepreneurial opportunities, however, often are discovered while being a paid employee acquiring knowledge about markets, customers and production technologies [63]. The second reason is related to the first reason. Because entrepreneurship is not yet an option for many, students at universities were not asked this question in the questionnaire. Thus, for 53% of the sample, no information about entrepreneurial behavior is available. While there are options to impute missing values, we feel not comfortable to impute that many missing values for a potential dependent variable which can bias our results. Instead we opted for using entrepreneurial intentions as our major dependent variable. Theory and empirical evidence argues that entrepreneurial intentions precede action [34,35,39]. In our data the correlation between intentions in wave 6 and entrepreneurial behavior in wave 7 ranges from 0.12 to 0.17 (depending on which of the three different measures of entrepreneurial intentions one uses). We also present a robustness check using entrepreneurial behavior as an outcome of entrepreneurial intentions.

As expected in a longitudinal survey, from the initial 1,321 participants 28% were lost due to attrition. Please note that this attrition rate is comparatively low for longitudinal studies [116,117]. We discuss the issue of attrition and consequences for our empirical strategy in the results section.

### Measures

Table 1 describes the measured variables in detail. This includes means, standard deviations, Cronbach’s alphas and sample items. In the following, we provide additional information on the variables.

**Table 1:** Description of the measured variables.

Variables/Scale/Source	Sample Item	Mean (*SD*)	Cronbach’s alpha^a^
**Entrepreneurial intentions** **(Age 26)** (36)			.89
**1) Item 1** (Scale: 1 to 7)	In the foreseeable future, do you intend to found a new business?	2.28 (*1*.*67*)	
**2) Item 2** (Scale: 1 to 7)	I have recently sought information about the ways and means of founding a new business.	1.92 (*1*.*65*)	
**3) Item 3** (Scale: 1 to 6)	In your opinion, how high is the probability that, in the foreseeable future, you will found a new business?	2.25 (*1*.*28*)	
**Skill variety (Age 26)** (13)			n.a.
**1) School interest variety** (Scale: 0 to 4)	Count of dummy variables of interest in/variety in subjects (scale: 1 to 7). Five school subjects: 1 = Mother tongue; 2 = Foreign language; 3 = Science; 4 = Humanistic and social sciences; 5 = Arts and handwork. Dummy: 1 = Rating greater than 3; 0 = Otherwise	2.25 (*1*.*22*)	
**2) Education** (Scale: 0 to 6)	Count of functional areas in which person has had educational/work experience. Six possible categories: 1 = General management; 2 = Sales, marketing, customer service; 3 = Finance, accounting; 4 = Technical, research, science, engineering; 5 = Manufacturing, operations; 6 = Administration, human resource management	2.55 (*1*.*68*)	
**3) Work** (Scale: 0 to 6)		2.16 (*1*.*51*)	
**Female (Age 16)**	Dummy: 1 = Female; 0 = Male	.54 (*0*.*50*)	n.a.
**Entrepreneurial parent (Age 16)**	Dummy: 1 = One/both parents self-employed, worker on own account/liberal profession; 0 = Otherwise	.09 (*0*.*28*)	n.a.
**SES (Age 16)** [118] (Scale: 1 to 3)	Socio-economic status of household: 1 = Blue collar to 5 = Upper white collar. Only highest-scoring result of parents is counted.	4.14 (*0*.*96*)	n.a.
**School track (A/B) (Age 16)**	Dummy: 1 = School track B; 0 = School track A	.46 (*0*.*50*)	n.a.

^a^ Cronbach’s alpha is only reported for true scales.

#### Dependent variable

To measure the dependent variable *entrepreneurial intentions* (assessed in T7, average age 26), participants were asked to answer three questions (item 1: “In the foreseeable future, do you intend to found a new business?”; 1 = Do not agree at all, 7 = Strongly agree; item 2: “I have recently sought information about the ways and means of founding a new business.”; 1 = Do not agree at all, 7 = Strongly agree; item 3: “In your opinion, how high is the probability that, in the foreseeable future, you will found a new business?”; 1 = 0%, 6 = 100%). The items have already been assessed in different studies [36,119].

#### Independent variable

*Female* serves as independent variable. We create a dichotomous variable where “1” represents being female and “0” denotes being male (assessed in T1, average age of 16 years).

#### Mediator

We measure skill variety in three different ways. First, we analyze skill variety at secondary school level (accessed in T1, average age of 16 years). Here, we calculate a variety index over different school subjects. At secondary school, all students have the same subjects. Thus, we cannot employ a variable covering different experiences or choices. But there is strong empirical evidence that interest in learning content leads to higher knowledge spillover and achievements in individual competencies [120,121]. Therefore, interest in subjects is a good proxy for learning outcomes (skills and knowledge).

The underlying school subjects are: (1) Mother tongue; (2) Foreign languages; (3) Science; (4) Humanistic and social sciences; (5) Arts and handwork (1 = Not at all interested, 7 = Interested very much). Please note that the categories mother tongue and foreign languages were coded into interest in languages in general. If the interest in a specific subject was greater than four, we created an auxiliary dummy variable with the value of one, otherwise the dummy variably was coded zero. The final variable *school interest variety* was computed by summing up the four corresponding dummy variables and ranges from zero to four.

Second, we analyze skill variety at university or vocational training level (tertiary education). Third, we analyze skill variety at work level. For both measures, we utilize the number of functional areas in which the participants have *educational* or *work* experience as an indicator for *skill variety* (accessed in T7, average age of 26 years). We created two independent count variables each underlying six possible categories: (1) General management; (2) Sales, marketing, customer service; (3) Finance, accounting; (4) Technical, research, science, engineering; (5) Manufacturing, operations; (6) Administration, human resource management. Thus, the variables range from zero to six. Similar measures have been employed successfully in previous research [9,13,76].

Although work experience is arguably the most important predictor of entrepreneurship [9], we consider it important to look at skill variety from a developmental perspective (secondary and tertiary education level), in when particular taking a gender perspective. Prior research shows that vocational differences between men and women grow over time, especially from middle school on [122]. Further arguments to include the developmental perspective stem from the sample. FinEdu is a longitudinal study that pursues the development from adolescence to young adulthood. At the 2014 wave the respondents are 26 years old. To some extent the respondents are still at school or at university (42%), which is quite typical for the Finnish educational system [123] but makes it somewhat unlikely for those to have skill variety in work. Other entrepreneurship studies have found curriculum variety to be an important predictor of future skill variety in work and entry into entrepreneurship [9,25].

#### Control variables

We had argued above that social capital and financial capital additionally contribute to the gender gap in entrepreneurship. Regarding social capital, we include *entrepreneurial parents* as control variable. Prior research has shown that skill variety [25] and entrepreneurship [124] strongly depend on the presence of self-employed parents. We assess *entrepreneurial parents* at T1 (average age of 16 years); with the value of one, if the respondent reported to have a father or a mother that is a 1) Self-employed person, 2) Worker on own account or 3) Freelancer in a liberal profession. With regard to financial capital within the family, we control for the family *socio-economic status (SES*, assessed at T1, average age of 16 years). The participants were asked to report their parents’ occupations. The answers were coded into five socio-economic categories, following a Finnish standard classification system [125]: 1 = Blue-collar (e.g. electrician, baker or hairdresser) to 5 = Upper white-collar (e.g. engineer, doctor or journalist). Parents who were not employed (e.g. students, pensioners or disabled), were coded as missing. Following [118], the parent with the highest occupation serves as reference for the SES. As explained above, we controlled for the affiliation to school track (0 = School track A, 1 = School track B).

## Results

### Empirical strategy

To test our hypotheses, we estimate structural equation models (SEM), utilizing Stata 12.1 [126]. SEM brings the advantage that we can examine different direct and indirect effects (mediation) in one model. Further, several fit indices make clear how well the data fits the conceptual model. We take χ2, CFI and RMSEA into account [127]. Last, SEM enables us to model latent variables, which are not affected by measurement errors. We model entrepreneurial intentions in our analysis as latent variables [36].

Recall that we observe some attrition (28%) from the first wave (T1) to the last wave (T7). Men had a higher probability to drop out of the study than women (χ2 = [1, N = 871] = 47.33, p < .001). Associated with this fact, there are also differences (men have lower scores) concerning *school interest variety* (t(1101) = 7.83, p < .1). The probability to drop out of the study is linked to the interest in different school subjects, which itself seems to be higher among women. These results are not surprising because the early waves of the study were conducted at school and school leavers were hard to follow. In view of the attrition, we imputed missing values by means of the “method(mlmv)” command in Stata 12.1 (maximum likelihood with missing values) [128]. Note that we also performed the analysis with a restricted sample (participants that were available through all waves), which did not yield substantially different results.

### Preliminary analyses

Correlation analysis and variance inflation factors (VIF) indicate that problems of multicollinearity are unlikely. While the mean VIF score is 1.10, the highest VIF score is 1.26 for *work skill variety*. The reported VIF is well below the recommended level of 10 [129]. Table 2 provides zero-order correlations between the manifest variables (Pearson). There is a significant correlation between *entrepreneurial intentions* and *skill variety* (education and work), *female* as well as an *entrepreneurial personality profile*. The third item measuring *entrepreneurial intentions* also shows a significant correlation to *entrepreneurial parents*. Each of the three variables measuring *skill variety* shows a significant correlation with *female* and an *entrepreneurial personality profile*. In addition, *educational* and *work skill variety* are significantly correlated with *children* and *fear of failure*, respectively. *Female* is significantly correlated to both *children* and *fear of failure*.

**Table 2:** Pearson correlations between the variables^a^.

		1	2	3	4	5	6	7	8	9	10
1.	Entrepr. intentions (item 1)	1.00									
2.	Entrepr. intentions (item 2)	0.73***	1.00								
3.	Entrepr. intentions (item 3)	0.84***	0.69***	1.00							
4.	School interest variety	0.01	0.05	0.05	1.00						
5.	Educational skill variety	0.18***	0.19***	0.20***	0.07**	1.00					
6.	Work skill variety	0.29***	0.26***	0.29***	0.00	0.39***	1.00				
7.	Female	-0.25***	-0.19***	-0.22***	0.18***	-0.08**	-0.19***	1.00			
8.	Entrepreneurial parent	0.05	0.05	0.08**	0.03	-0.01	0.04	0.03	1.00		
9.	SES	-0.04	-0.01	-0.02	0.07**	0.00	-0.03	0.02	-0.17***	1.00	
10.	School track	-0.12***	-0.08**	-0.11***	0.16***	0.02	-0.08**	0.14***	0.04	0.09***	1.00

^a^ *p < .05

**p < .01

***p < .001.

### Hypotheses testing

#### The direct effect of gender on entrepreneurial intentions

**Fig 1:**
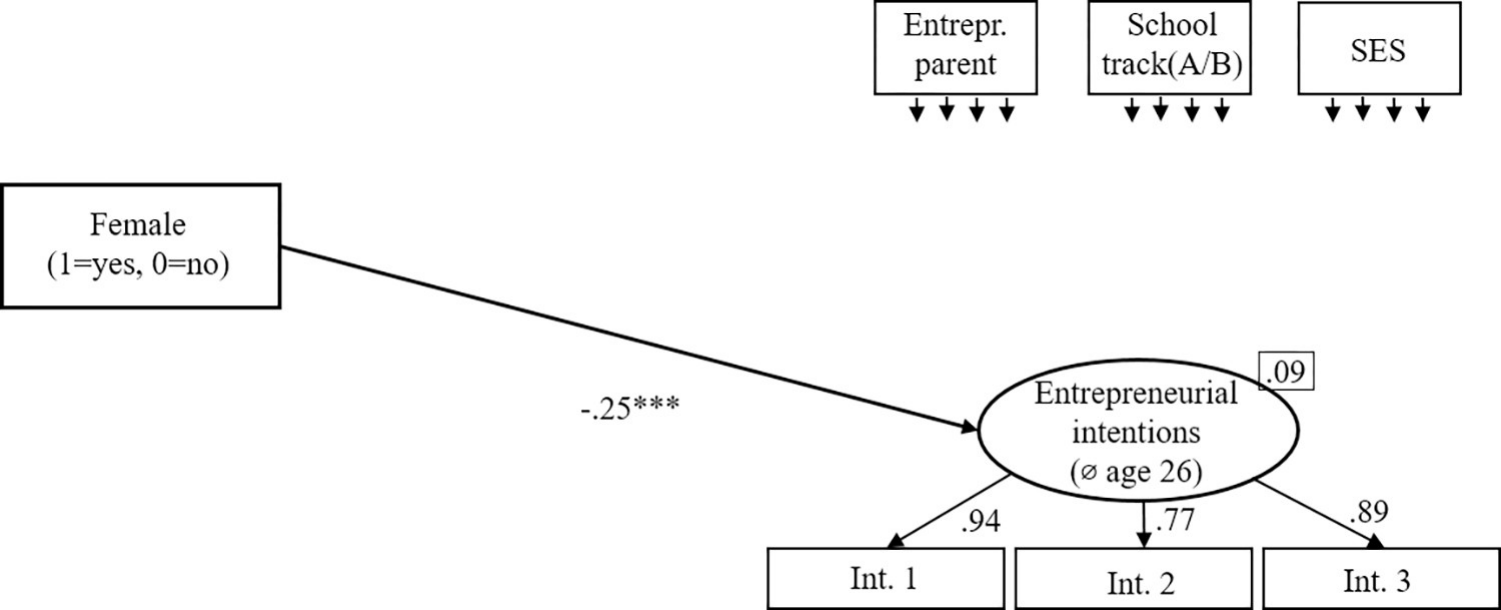
The gender gap in entrepreneurial intentions: Direct effects of gender on entrepreneurial intentions in adulthood. Standardized coefficients are given. *R^2^* is shown in the upper right corner of the dependent variable. Correlations between the control variables were allowed. *p < .05; **p < .01; ***p < .001.

To test the baseline hypothesis, stating that being female negatively predicts entrepreneurial intentions, we set up a structural equation model (Fig 1) that controls for entrepreneurial parents and SES. The model shows an excellent fit (χ2[8] = 5.27, p = .729, CFI = 1.000, RMSEA = .000). Being female predicts entrepreneurial intentions with a negative effect of β = -.25 (p < .001). Hence, H1 receives full support.

#### Skill variety as a mediator

**Fig 2:**
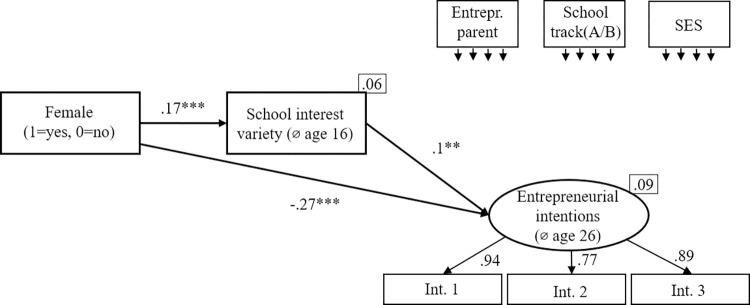
Early variety (school interest variety at age 16) as mediator. Standardized coefficients are given. *R^2^* is shown in the upper right corner of the dependent variable. Correlations between the control variables were allowed. *p < .05; **p < .01; ***p < .001.

**Fig 3:**
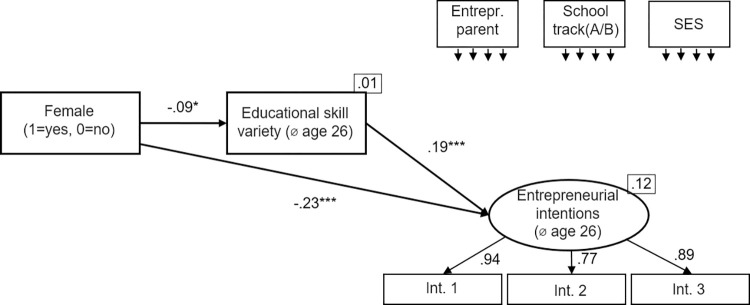
Educational skill variety as mediator. Standardized coefficients are given. *R^2^* is shown in the upper right corner of the dependent variables. Correlations between the control variables were allowed. *p < .05; **p < .01; ***p < .001.

**Fig 4:**
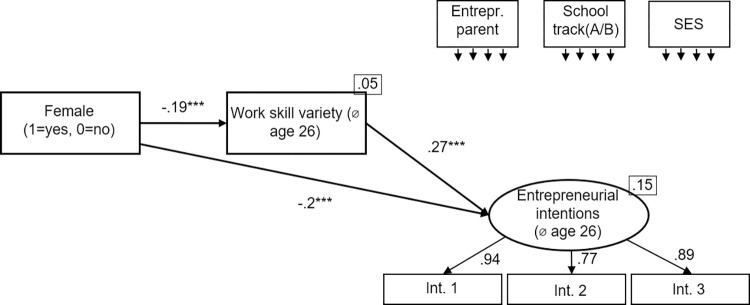
Work skill variety as mediator. Standardized coefficients are given. *R^2^* is shown in the upper right corner of the dependent variables. Correlations between the control variables were allowed. *p < .05; **p < .01; ***p < .001.

We use three different measures for skill variety (*school interest variety*, age 16; *educational skill variety*, *age 26; work skill variety*, *age 26*). In a first step, we look at the different measures for skill variety in separate models (Figs 2–4). Fig 2 shows the mediation model using *school interest variety* as measure for skill variety. Fig 3 shows the mediation model using *educational skill variety* as measure for skill variety. Fig 4 shows the mediation model using *work skill variety* as measure for skill variety. All models show an excellent fit (Fig 2: χ2[10] = 10.14, p = .428, CFI = 1.000, RMSEA = .000; Fig 3: χ2[10] = 9.06, p = .523, CFI = 1.000, RMSEA = .000; Fig 4: χ2[10] = 6.19, p = .80, CFI = 1.000, RMSEA = .000).

In regard to the second hypothesis, stating that being female negatively predicts skill variety, we found mixed evidence over the three models. Being *female* did not negatively predict *school interest variety*; in contrast, there is a positive relation (β = .17, p < .001). But being *female* negatively predicted both *educational skill variety (*β = -.09, p < .01) and *work skill variety* (β = -.19, p < .001). Hence, H2 receives partial support over the models.

To test the third hypothesis, stating that skill variety positively predicts entrepreneurial intentions, we also used the three models described above. All three skill variety measures predict entrepreneurial intentions (*school interest variety*: β = .1, p < .01; *educational skill variety*: β = .19, p < .001; *work skill variety*: β = .27, p < .001). Hence, H3 received full support over the models (consistent with the earlier research [14]).

To test the fourth hypothesis, stating that the relationship between being *female* and *entrepreneurial intentions* is mediated by *skill variety*, we used the three models described above and applied bootstrapping with 2,000 replications. For each of 2,000 bootstrapped samples the unstandardized indirect effects and the 95% confidence intervals were computed by determining the indirect effects at the 2.5th and 97.5th percentiles. The mediation results are summarized in Table 3. First, the standardized indirect effect of being *female* over *school interest variety* on *entrepreneurial intentions* was β = .02. The unstandardized bootstrapped effect was .04, with 95% confidence intervals of .01 to .07. Even though the indirect effect was statistically significant, the effect works the other way round than expected.

**Table 3:** Mediation effects.

Relationship	Female → Entrepreneurial intentions^a^^,^^b^
Direct effect without mediators	-.25***
Direct effect with mediator: School interest variety	-.27***
Indirect effect with mediator: School interest variety	.02** (.04) [.01 to .07]
Direct effect with mediator: Educational skill variety	-.23***
Indirect effect with mediator: Educational skill variety	-.02** (-.04) [-.07 to -.00]
Direct effect with mediator: Work skill variety	-.2***
Indirect effect with mediator: Work skill variety	-.05***(-.12) [-.17 to -.07]

^a^ Standardized effects are given (unstandardized effects in parenthesis). Indirect effects and confidence intervals (95% CI reported in squared brackets) were estimated with 2,000 bootstrap resamples. Unstandardized confidence intervals are reported. All effects are controlled for entrepreneurial parents, SES and school track.

^b^ *p < .05

**p < .01

***p < .001.

Second, the standardized indirect effect of being *female* over *educational skill variety* on *entrepreneurial intentions* was β = -.02. The unstandardized bootstrapped effect was -.04, with 95% confidence intervals of -.07 to -.00. Thus, the indirect effect was statistically significant. Because we still observe a significant negative effect of being *female* on *entrepreneurial intentions*, this suggests partial mediation. Third, the standardized indirect effect of being *female* over *work skill variety* on *entrepreneurial intentions* was β = -.05. The unstandardized bootstrapped effect was -.12, with 95% confidence intervals of -.17 to -.07. Thus, the indirect effect was statistically significant, again suggesting partial mediation. Hence, H4 received support.

#### Comparison of the skill variety measures across the educational trajectory

**Fig 5:**
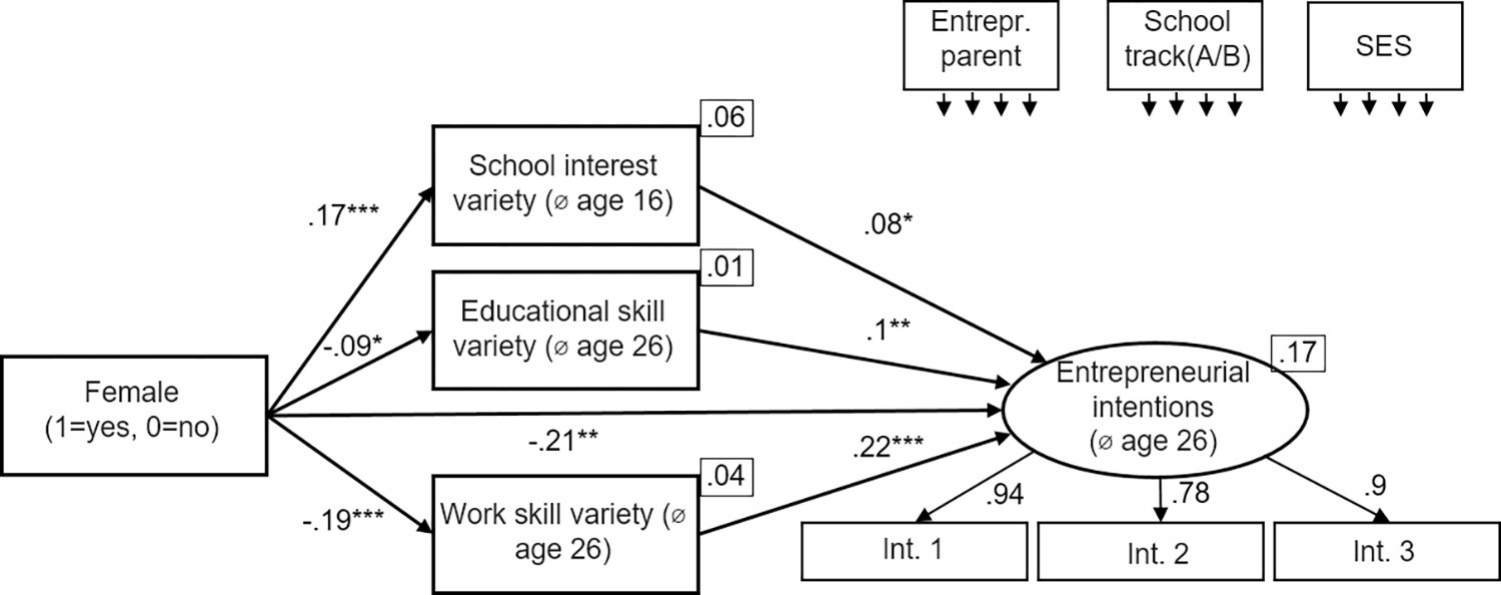
Seemingly unrelated regression model. Standardized coefficients are given. *R^2^* is shown in the upper right corner of the dependent variables. Correlations between the control variables and between the mediators were allowed. *p < .05; **p < .01; ***p < .001.

Hypothesis 5 stated that the negative indirect effect of gender via skill variety on entrepreneurial intentions grows stronger across the educational trajectory, being weakest when using *school interest variety* and being strongest when looking at *work skill variety*. The indirect effects reported above (β = .02 for school interest variety, β = -.02 for educational skill variety and β = -.05 for work skill variety) can be regarded as first empirical evidence in favor of this hypothesis. However, this initial comparison is only an eye-test. For an econometrically sound comparison of the regression coefficients we follow the recommendations of previous research [130,131] to test the equality of regression coefficients. Using SEM, the three models described above are estimated simultaneously and the coefficients are compared afterwards (seemingly unrelated regression). This simultaneously estimated model is presented in Fig 5. Please note that the error terms are assumed to be correlated across the different measures for *skill variety*. This procedure yields the correct coefficients and their standard errors. The coefficients represent the point estimators. For a comparison of whether these estimated regression coefficients are different, we then determine the difference between the coefficients and see if this difference is greater than a certain limit value. This procedure corresponds to an χ2-test. The comparison in Fig 5 shows an excellent fit (χ2[14] = 13.58, p = .482, CFI = 1.000, RMSEA = .000).

First, we present the results of the comparison of the relationship between being *female* and the three *skill variety* variables. The negative effect of being *female* on *educational skill variety* (β = -.09, SE = .03) is stronger than the effect of being *female* on v*ariety interest in subjects* (β = .17, SE = .03, χ2 = 33.2, p < .001. Furthermore, the negative effect of being *female* on *work skill variety* (β = -.19, SE = .03) is stronger than the effect of being *female* on *variety interest in subjects* (β = .17, SE = .03, χ2 = 66.8, p < .001) as well as stronger than the effect of being *female* on *educational skill variety* (β = -.09, SE = .03, χ2 = 7.77, p < .01).

Second, we compare the relation between the three different measures of *skill variety* and *entrepreneurial intentions*. Two out of three comparisons show significant differences. The positive effect of *educational skill variety* on *entrepreneurial intentions* (β = .1, SE = .04) is not significantly stronger than the effect of *school interest variety* on *entrepreneurial intentions* (β = .08, SE = .04, χ2 = 0.13, p>.1). In contrast, the positive effect of *work skill variety* on *entrepreneurial intentions* (β = .22, SE = .04) is significantly stronger than the effect of *school interest variety* on *entrepreneurial intentions* (β = .08, SE = .04, χ2 = 7.99, p < .01) as well as stronger than the effect of *educational skill variety* on *entrepreneurial intentions* (β = .1, SE = .04, χ2 = 4.52, p < .05).

Third, we compare the indirect effects within the comparison model. The procedure applied corresponds the one used above. Please note that the indirect effects and their standard errors were computed with Bootstrapping, using 2,000 replications. All three indirect effects were significant at 5%-level. We do not provide further details on these bootstrapping results, because we do not observe differences in comparison to the indirect effects within the models. In the following, we report unstandardized coefficients and standard errors as we are only interested in differences between the indirect effects rather than the effects themselves. The mediating effect of *female* via *educational skill variety* on *entrepreneurial intentions* (β = -.02, SE = .001) is significantly stronger than the mediated effect of *female* via *school interest variety* on *entrepreneurial intentions* (β = .03, SE = .012, χ2 = -354.9, p<0.001). The mediated effect of *female* via *work skill variety* on *entrepreneurial intentions* (β = -.1, SE = .034) is significantly stronger than the mediated effect of *female* via *school interest variety* on *entrepreneurial intentions* (β = .03, SE = .012, χ2 = -129.8, p < .001 as well as stronger than the mediated effect of *female* via *educational skill variety* on *entrepreneurial intentions* (β = -.02, SE = .001, χ2 = -70.6, p<0.001). Taken together, we conclude Hypothesis 5 to be supported.

**Fig 6:**
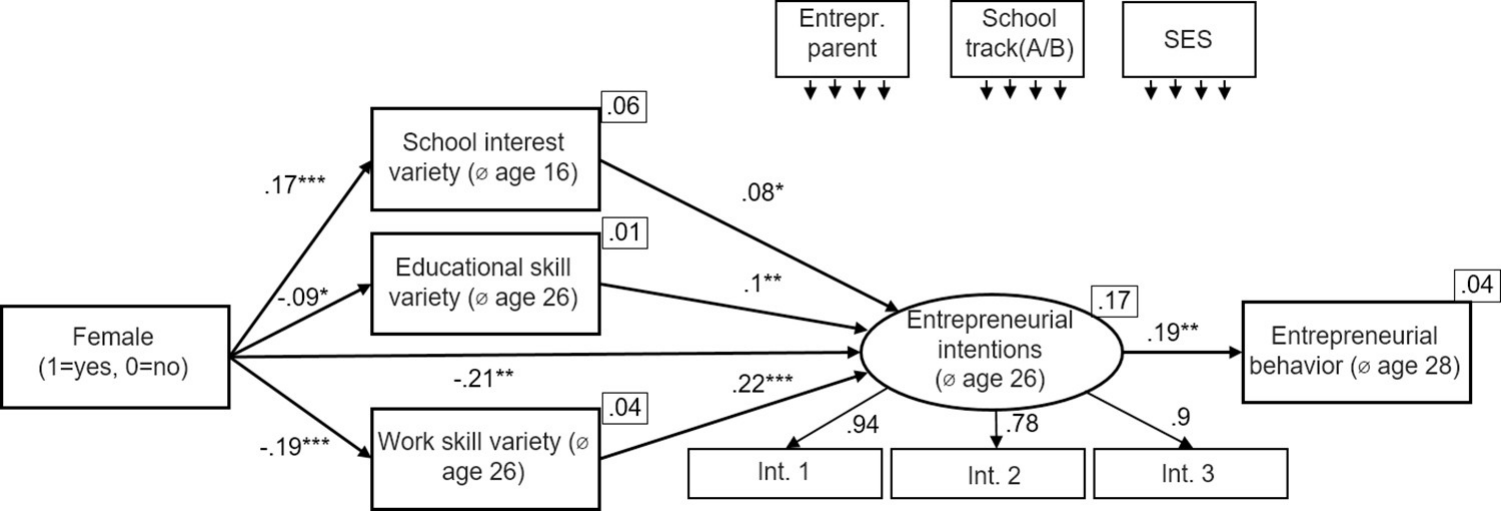
Seemingly unrelated regression model with entrepreneurial behavior succeeding entrepreneurial intentions. Standardized coefficients are given. *R^2^* is shown in the upper right corner of the dependent variables. Correlations between the control variables and between the mediators were allowed. *p < .05; **p < .01; ***p < .001.

In a robustness check, we present in Fig 6 a mediation model using *entrepreneurial behavior* as final outcome succeeding entrepreneurial intentions. Thereby, *entrepreneurial behavior* is a dummy variable taking the value of 1 if the respondent had already founded a business which he or she has owned fully or partly. *Entrepreneurial intentions* predict *entrepreneurial behavior* (β = .19, p < .001). All other path coefficients remain the same compared to Fig 5. Thus, all hypotheses remain supported.

In the SEM in Fig 5, the skill variety measures only predict *entrepreneurial intentions* but not *entrepreneurial behavior*. Note, that the results not change if we alternatively use the skill variety measures as additional predictors for entrepreneurial behavior. The model (not shown here because of brevity) reveals that the skill variety measures are bad predictors of *entrepreneurial behavior* in our sample with only *work skill variety* significantly predicting behavior at the 10% significance level. However, the relationships of the skill variety measure with *entrepreneurial intentions* remain. These results support our reasoning for using intentions instead of action as dependent variable in our main models.

## Discussion

### Summary of main results

The aim of this paper was to investigate the gender gap in entrepreneurship from a skill variety perspective. Drawing on human capital theory [61], the skill variety approach [9] and research on differences in labor market participation, we theorized on the differences in skill variety between the sexes. We focused on two research questions. First, to what degree does skill variety provide an explanation (as a mediator) for the gender gap in entrepreneurial intentions? Second, how does the mediating effect of skill variety in the relationship between gender and entrepreneurial intention change over the educational trajectory? We observe a gender gap in *entrepreneurial intentions* (age of 26 years). Looking at the relation between *female* and *skill variety*, we find mixed evidence. *School interest variety* (age of 16 years) showed a comparably high positive connection with being *female*. However, the other two measures of skill variety, *educational* and *work skill variety* (both age of 26 years), were, as hypothesized, negatively associated with being *female*.

Results from bootstrapping showed significant negative indirect effects from being *female* over *educational* and *work skill variety* on *entrepreneurial intentions*. Interestingly, the indirect effect from being *female* over *school interest variety* on *entrepreneurial intentions* is positive because women show more variety over different school subjects. The formation of subsequent variety in skills might be a development that is triggered by the gender-typical occupational choice and socialization, respectively discrimination in the labor market. For example, women might be both attracted and forced into more specialized jobs (often in the public sector). Results from a seemingly unrelated regression analysis showed that the closer the skill variety measure to the labor market, the stronger the negative gender effect is. Thus, the negative indirect effect from being *female* over *work skill variety on entrepreneurial intentions* is stronger than the negative indirect effect over *educational skill variety*.

### Discussion of the results

The results of our paper can inform the more general research on gender and entrepreneurship. Human capital differences between both genders had been discussed as a source of the gender gap in entrepreneurship over the last decades but with shrinking intensity. For instance, the review of the female entrepreneurship literature by Poggesi et al. [132] mentions some education and work experience variables only peripheral and never even mentions the term human capital. Moreover, the annotated bibliography on gender and entrepreneurship of Link and Strong [133] list only 10 papers from the year 2010 on referenced as human capital papers. Our results suggest that there are systematic gender differences in skill variety, widely regarded as the most promising advancement in human capital theory for entrepreneurship, and that these differences matter. This echoes the call for more research by Link and Strong [133] on categories and types of education and experience that matter for entrepreneurship. In a related topic, Strohmeyer et al. [32] provide empirical evidence that gender differences in innovativeness can be attributed to skill variety differences.

Our paper also provides some empirical evidence that these skill variety differences do not suddenly appear out of thin air but develop over time. Thus, our paper contributes to research investigating the origins and development of (entrepreneurial) human capital [13,134,135], and to research investigating the sources of human capital differences between the genders [4,72,136]. We found that women *do not* have less variety in school, quite the opposite as they have more compared to men. The data suggests that the gap in variety, with women scoring lower than men, starts to open up in tertiary education and widens at the labor market. Our results do support recent findings from Tonoyan et al. [4] who showed that the combination of woman working in non-managerial positions, in female dominated occupations and sectors explain a substantial share in the perceived ease to start a business. It is arguably the differences in skill variety, that establish the mediating channel between these work experiences and lower entrepreneurial intentions for woman. Our results do not support the findings from Lavy and Sand [136] that show gender differences in scores in difference subjects as early as in secondary education. It is however important to note that our data stem from Finland which is country with a relatively high level of gender equality in both public and private life.

Regarding the context of the study in Finland, it is likely that one would observe different patterns in different countries as factors such as economic development and culture play a substantial role in the formation of occupational and entrepreneurial aspirations and its materialization in entrepreneurial action [137,138]. We note however that the present results are counter-intuitive. Finland as a country with strong female empowerment still shows substantial gender differences in educational and occupational trajectories, while one might expect the opposite. However, our results are well in line with recent findings from cross-country comparisons of occupational aspirations, where Stoet and Gary [139] report that gender-typical occupational aspirations (such as boys aspiring things-oriented occupations and girls aspire people-oriented occupations) is larger in countries with strong female empowerment such as in the developed North European countries.

### Limitations and future research

This paper has several limitations. First, we acknowledge that the effect sizes of the skill variety measures in explaining the gender gap in entrepreneurial intentions are relatively small. *Educational skill variety* explains approximately 8%, while *work skill variety* explains 20% of the gender gap. The gender gap is a multi-causal phenomenon (1). Future research could thus include a broader set of gender typical explanatory variables from social and financial capital. Future research could also explore the conditions under which being female actually leads to less skill variety. The role of the socio-economic environment, culture or micro-level indicators (e.g. entrepreneurial parents) might be important drivers or inhibitors of female entrepreneurship. With regard to the measurement of skill variety, in a gender context, it might be an interesting idea for future research, to measure movement around firms and industries. From this mobility, chances to acquire skill variety arise. Women might be particularly disadvantaged here, because of their reconciliation of family and work as well as their higher risk aversion.

Second, we mainly use entrepreneurial intentions and not behavior as outcome variable. The participants of the FinEdu study were only 28 years old at the last wave of data collection. Not all respondents have completed the transition into working life yet. Thus, we argue that it was too early to assess entrepreneurial actions and thus decided for entrepreneurial intentions as outcome. Previous research has shown that entrepreneurial intentions, measured in adolescence, predict subsequent entrepreneurial activity [39]. Beyond that, we also presented a robustness check using entrepreneurial behavior as outcome succeeding entrepreneurial intentions which similar results.

Furthermore, one main limitation is that this study was conducted in Finland and the results thus cannot be generalized to other countries easily. Finland is a welfare state, both in terms of education and social system. The data was raised for only two different types of school, it might be more useful to investigate participants with more heterogeneous educational backgrounds. Furthermore, it might be interesting to replicate the results for countries with different institutional frameworks or formal rules (e.g. concerning child care or household obligations). With a look at the issue of attrition, we cannot be sure whether those participants who dropped out of the study differed from those who lasted till the end, in terms of skill variety or entrepreneurial intentions. A clear limitation is that male participants were more likely to drop out than female participants. Although in this school setting of data collection this is reasonable, in particular for a gender discussion a more balanced attrition would have been desirable.

Regarding future research, our study is silent on the actual mechanism how educational and occupational choices ultimately result in lower skill variety for women. Our theorizing centered around part-time work, working fewer years, working in rank-and-file positions, studying and working in the public sector for various reasons including discrimination, family commitments and traditional gender roles. However, our dataset does not allow testing the importance of these different channels. This is left for future research.

### Implications for practice

This paper has important implications for policy makers and entrepreneurship educators. Skill variety is important for entrepreneurial choice, success and entrepreneurship in general. If women ultimately develop less entrepreneurial skill variety than men, as indicated in our study, policy makers should then empower women and facilitate investments in such skill variety underlying entrepreneurial intentions, behavior, and success. This should also focus on potential barriers preventing women from growing the diversity of their skills. Hence, politics should not only create institutional environments and entrepreneurship programs which provide women with opportunities and incentives to acquire skill variety, but should also eliminate potential, existing barriers in the development of entrepreneurial human capital. Interestingly, our results indicate that in adolescents women even outperform men with respect to early representations of skill variety (school-related skill variety). This indicates that the gender gap in entrepreneurial skill variety, where females fall behind, gets imprinted later (e.g., in tertiary education and the early vocational career). This crucial turning point, where women fall behind, should be the main focus of intervention programs designed to level the playing field–to empower women in their development of crucial entrepreneurial human capital by addressing the actual roots of the gender gap in entrepreneurship. However, we note in the light of Stoet and Gary (2022) that even high empowerment of woman and the development of training programs for girls and women, will not completely close the gender gap in entrepreneurship because the still existing self-selection in gender-typical occupations remain a powerful driver across the educational and occupational trajectory.
